# Perimeter Intrusion Detection by Video Surveillance: A Survey

**DOI:** 10.3390/s22093601

**Published:** 2022-05-09

**Authors:** Devashish Lohani, Carlos Crispim-Junior, Quentin Barthélemy, Sarah Bertrand, Lionel Robinault, Laure Tougne Rodet

**Affiliations:** 1 Univ Lyon, Univ Lyon 2, CNRS, INSA Lyon, UCBL, LIRIS, UMR5205, F-69676 Bron, France; carlos.crispim-junior@liris.cnrs.fr (C.C.-J.); lionel.robinault@liris.cnrs.fr (L.R.); laure.tougne@liris.cnrs.fr (L.T.R.); 2 Foxstream, F-69120 Vaulx-en-Velin, France; q.barthelemy@foxstream.fr (Q.B.); s.bertrand@foxstream.fr (S.B.)

**Keywords:** perimeter intrusion detection, video surveillance, outdoor environment, real-time analysis, i-LIDS

## Abstract

In recent times, we have seen a massive rise in vision-based applications, such as video anomaly detection, motion detection, object tracking, people counting, etc. Most of these tasks are well defined, with a clear idea of the goal, along with proper datasets and evaluation procedures. However, perimeter intrusion detection (PID), which is one of the major tasks in visual surveillance, still needs to be formally defined. A perimeter intrusion detection system (PIDS) aims to detect the presence of an unauthorized object in a protected outdoor site during a certain time. Existing works vaguely define a PIDS, and this has a direct impact on the evaluation of methods. In this paper, we mathematically define it. We review the existing methods, datasets and evaluation protocols based on this definition. Furthermore, we provide a suitable evaluation protocol for real-life application. Finally, we evaluate the existing systems on available datasets using different evaluation schemes and metrics.

## 1. Introduction

In the last two decades, we have seen tremendous innovation in vision-based systems [1]. The massive installations of cameras in almost all essential sites, from banks to supermarkets and in prominent streets, have further helped in developing and testing these systems. Visual surveillance is one of the most important and relevant domains for intelligent vision systems [2]. Visually surveying a site can include various tasks, such as object detection, object tracking and anomalous behaviour detection [3]. One such task is perimeter intrusion detection (PID), which aims to detect the presence of an unauthorized object in a protected outdoor site during a certain time [4,5,6]. The cameras record videos continuously in the outdoor site to be protected. The fact that it is an outdoor environment is very important here as it comes with challenges such as changing weather and light conditions, insects, animals, etc., contrary to an indoor environment [7,8]. The user defines protection area on the scene, potential intruder objects and the time during which the system needs to protect (e.g., protection during night only). Given user needs, the perimeter intrusion detection system (PIDS) detects intrusion and sends an alarm signal to the surveillance personnel for verification.

**Figure 1:**
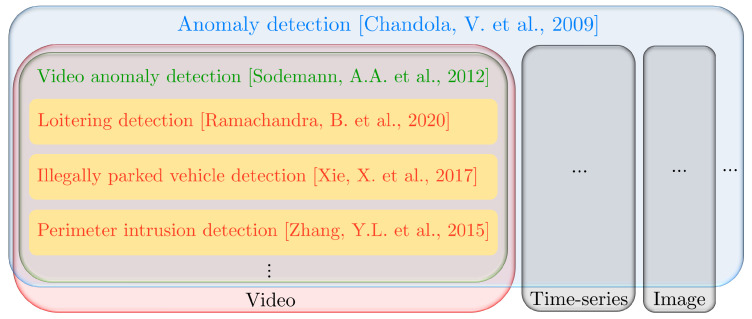
Venn diagram to illustrate the taxonomy of tasks in video anomaly detection.

One of the key goals of video surveillance is to detect behaviours that can be considered anomalous. Anomalies are patterns in data that do not follow a well-defined notion of normal behaviour [9]. Depending on the nature of input data and context, anomalies can refer to different patterns, such as abnormal sections in a time-series data, abnormal patches in an image, abnormal spatio-temporal volumes in a video, etc., as illustrated in Figure 1. Concerning video data, video anomaly detection [10,11] refers to the detection of unusual appearance or motion attributes in the video. In [12], a dataset is proposed containing 13 anomalous activities, such as abuse, arrest, accident, explosion, etc., and they used multiple instance learning to detect anomalies. Depending on the context, video anomaly detection can be specified in different tasks, such as abandoned object detection [13], loitering detection [14], illegally parked vehicle detection [15], etc. Perimeter intrusion detection also falls into this category [16,17,18]. In fact, intrusions are a particular type of anomalies, classified as point and contextual anomalies by (Chandola et al. [9], Section 3.5). Moreover, the notions of perimeter, intruder movement and site protection time are crucial for the PID task, i.e., anomalous/unauthorized objects present in the video are intruders only if they are in movement inside the designated perimeter when the site is being surveyed. In other words, all intrusions are anomalies but not all anomalies are intrusions.

In the visual surveillance literature, we find several comprehensive reviews on various tasks, such as object detection [19,20], object tracking [21], anomaly detection [9], etc. However, the PID task lacks such a review work. Few works define the PID task, but their definition requires clarity [4,22,23]. It is essential to mathematically define the task as its definition has a direct impact on the evaluation. In practice, when the PIDS detects an intrusion, it sends a short video to the security post, where a human operator validates the alarm as a true intrusion or otherwise. This short video, composed of several frames before and after the suspected intrusion, must contain the intruder so that the operator can decide to send the security team to the perimeter. The end of the intrusion event is not relevant for this application. Consequently, the task of PID by video can be seen as the detection of the beginning of an abnormal event in a perimeter. In the PID task, we want to detect intrusion caused by human-based activities such as walking, driving a car, etc. To be sure to not miss such an intrusion, the video must be acquired at between 5 and 25 frames per second (FPS) [24]. This is the real-time constraint of this task. In practice, we would like to detect intrusion as soon as it occurs; thus, we have time constraints. This requires a suitable evaluation protocol that takes these particularities into account.

The intrusion detection task is closely related to other surveillance tasks and many of these tasks, such as motion detection and tracking, can be an essential part of a PIDS pipeline. Many existing methods address one of these auxiliary tasks in the surveillance system. Only few methods tackle the problem of PIDS completely [22,24,25]. Since missing intrusions in a site is considered as a major failure for a PIDS, existing methods are optimized to detect as much as possible even at the cost of some false alarms [24]. Similarly, unlike for auxiliary tasks, there is no standard protocol for evaluating a PIDS. The dataset i-LIDS defines an evaluation protocol [26] but it is not widely adopted and has several drawbacks, which we detail in Section 5.2.

Our main contributions are summarized as follows: (i) We propose a formal definition of the PID task; (ii) We review the existing methods, datasets and evaluation protocols; (iii) We provide a novel PID evaluation protocol; (iv) We compare all evaluation protocols on a common dataset using existing methods.

This article is organized as follows. In Section 2, we review the PID data with different data acquisition strategies, their challenges and available datasets. Existing PID methods are presented in Section 3. Section 4 formalizes the PID task using mathematical definitions. In Section 5, we explain various evaluation protocols/metrics and present a new evaluation protocol. In Section 6, we compare the evaluation protocols using existing methods. We next provide a discussion in Section 7. Finally, we conclude in Section 8.

## 2. Review of PID Data

In this section, we first describe the various data acquisition systems with their associated advantages and drawbacks. Then, we identify the challenges associated with a PIDS. Finally, we detail the existing datasets.

### 2.1. Data Acquisition

The area to be protected is observed with the help of cameras. These cameras acquire the video stream in order to detect possible intrusions. The acquired data can be used as an image sequence or as a video, depending on the system. The nature of data depends on the type of camera used. Broadly, the following categories of video capture devices are used.

#### 2.1.1. Visual Camera

These cameras capture the visible light in grey-scale or RGB images. Their advantage is that they render an image visually closer to the naked eye. However, they need a certain level of brightness in the scene and are sensitive to illumination changes [1,27]. At night, they need additional lighting for the sensor to restore a sufficiently contrasted image [22,28]. Adverse weather conditions such as fog, rain, snow, etc., further limit observation of objects to a short distance from the camera [22], and thus make detection difficult. Even after all of these drawbacks, these cameras have been used extensively in video surveillance systems as they are the standard imaging device [3,29]. They are one of the cheapest available cameras.

#### 2.1.2. Infrared Camera

Infrared cameras capture near-infrared emissions [30] from objects and are suitable for environments with a low illumination level. They are coupled with infrared lighting that can provide better contrast when an object moves past the camera [31]. It is difficult to detect the object during rain in this camera as rain drops appear as thick stripes in front of the camera [28]. In addition, this camera attracts flying insects and spiders that can raise false alarms and, as a consequence, impact detection. These cameras are more expensive than the color/visual cameras, with typical costs 1.5 to 2 times more than the color cameras.

#### 2.1.3. Thermal Camera

Thermal cameras have passive sensors that capture the mid-wavelength infrared radiation emitted by all objects with a temperature above absolute zero [30]. The main advantages include no external lighting requirement [30], a lower sensibility to weather conditions, ignoring object shadows [28] and long-range detection [32,33]. However, the main drawback of the thermal camera is that it is difficult to distinguish an object from its background when both of them have almost the same temperature. Thermal cameras cost at least six times more than the color cameras.

#### 2.1.4. Other Types of Acquisitions

All of these camera types can be used with additional sensors. The depth information can help in determining the perspective size of an object and, thus, can help in better intrusion detection [8,23]. However, using a depth sensor also has several problems, such as mixed, lost and noisy pixels in the depth image [34,35].

Another type of camera, known as the event camera, captures only the motion information in the scene [36]. It finds its application in motion detection, object segmentation, pose estimation, motion tracking, etc. [37]. It cannot capture static motion and absolute intensity, and therefore is often used together with other cameras types. Event cameras are not used for tasks such as video anomaly detection and perimeter intrusion detection yet, where both spatial and temporal information are essential. The main reasons for this are its extremely high cost and inability to capture visual features, such as texture and color.

We can also have a multi-camera system with similar or different types of cameras. Intuitively, combining multiple sensors will provide more accurate information about the targeted object and help to overcome occlusion. A visual surveillance system using multiple cameras has been studied extensively [19,38,39].

Recently, an intelligent PIDS was proposed using an integrated image acquisition device that combines visual and thermal cameras [22]. However, a multi-camera system also brings new challenges, such as camera installation, camera calibration, object matching, automated camera switching and data fusion [19].

### 2.2. Open Challenges

In the context of PIDS, the cameras are generally fixed in a static position to monitor the area to be protected [3,40]. These areas include important industrial sites, private property or land, etc. [22]. Most of the time, little or nothing is moving in these sites. Some animals may move or trees may shake due to wind in the area but, generally, they should be ignored by the system. However, the system must be operational continuously for a number of days and encounter changing light and weather [26]. Detection of the intrusion must be carried out quickly, within a few frames after the intruder enters the scene [24,26,41]. Therefore, PID algorithms must address this type of scenario.

In a video surveillance context, a certain number of situations make intrusion detection difficult. Several authors [40,42,43] have identified different challenges. These challenges have been classified in different categories on the basis of data acquisition, scene capturing and the object of interest. We advise the reader to refer to the previously cited articles for more details.

### 2.3. Datasets

Since the task of intrusion detection can include various subtasks such as detection and tracking, historically algorithms were tested on the datasets of these subtasks. For example, CAVIAR [44], PETS2006 [45] and AVSS2007 [46] have been used to test the tracking module of PIDS [47,48,49]. However, even after the immense success of deep learning and computer vision in recent years, there is still only one dataset that is dedicated for intrusion detection: the i-LIDS sterile zone dataset [26]. It has been extensively used in the literature [4,24,25,41,48].

Other than i-LIDS, most works are on private datasets. One recent work introduces a new dataset called SIC [24], but it is available under strict conditions and, without annotations, omissions cannot be evaluated.

#### i-LIDS Sterile Zone Dataset

The imagery library for the intelligent detection systems (i-LIDS) sterile zone dataset has been published by the UK Home Office for the PID task [26]. It is carefully designed by end users of the technology to benchmark surveillance systems. It is provided with a clear problem definition, annotation and evaluation procedure (see Section 5.2) to ensure relevance for industrial application.

**Figure 2:**
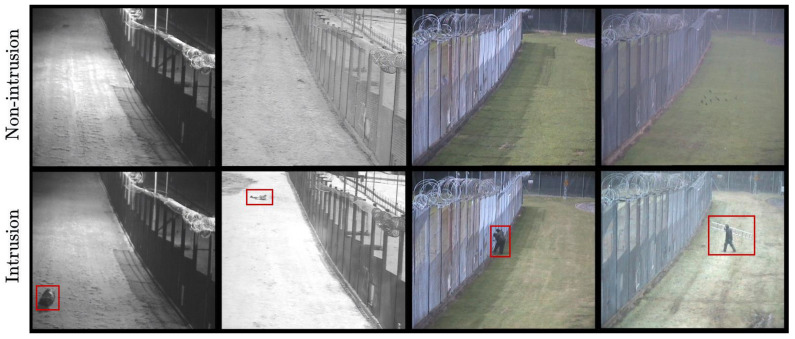
Some raw frames drawn from i-LIDS sterile zone dataset [26] with various intrusion (intruders in red boxes) and non-intrusion frames. The black and white and color frames belong to view 2 and view 1, respectively. The time of the day in four columns are night, day, dawn and dusk. The distractions in non-intrusion row (left to right order) are fox, insect on camera, shadow on fence and birds with rain, respectively. In intrusion row (left to right order), we have log rolling intruder, crawling intruder, two intruders and intruder with ladder during snow, respectively.

The PID task in this dataset consists of detecting the presence of people in a sterile zone. There are two sites monitored by two different cameras (view 1 in color/monochrome and view 2 in monochrome) as shown in Figure 2. Each site (view) is protected by a security fence and the aim is to detect intrusion before it passes through the fence. Intruders are one or two people trying to breach the fence in various ways. For example, people may walk, run, crawl or roll towards the fence and, on occasion, may be carrying climbing aids, such as a ladder. The intruders are situated at three different distances from the camera: close, middle and far. The cameras record videos over many days, capturing different times of the day, such as dawn, day, dusk and night. They further include various weather conditions, such as cloudy, rainy, snowy and foggy. Apart from intrusions, there are various distractions that might trigger false alerts. Possible distractions include plastic or paper waste moving due to wind, bats, birds, foxes, insects, rabbits, squirrels, shadows through the fence, etc. Figure 2 illustrates this with various examples. These different weather conditions, times of the day, various distractions and numerous ways of the intruder approaching the fence make it a very challenging and realistic dataset.

The dataset is divided into two disks for training and testing. The train disk is for developing the PIDS and the test disk for verifying its performance. Each disk has two cameras views with over 20 h of video recorded in the various previously cited situations. All videos are taken at 25 FPS with 720 × 576 frame size resolution. The annotation provided is the time interval of each intrusion event in the video, i.e., the entry and exit time of people in the respective scene. Table 1 summarizes the i-LIDS dataset with a number of videos and a different intrusion count per-video.

## 3. PID Methods

**Figure 3:**
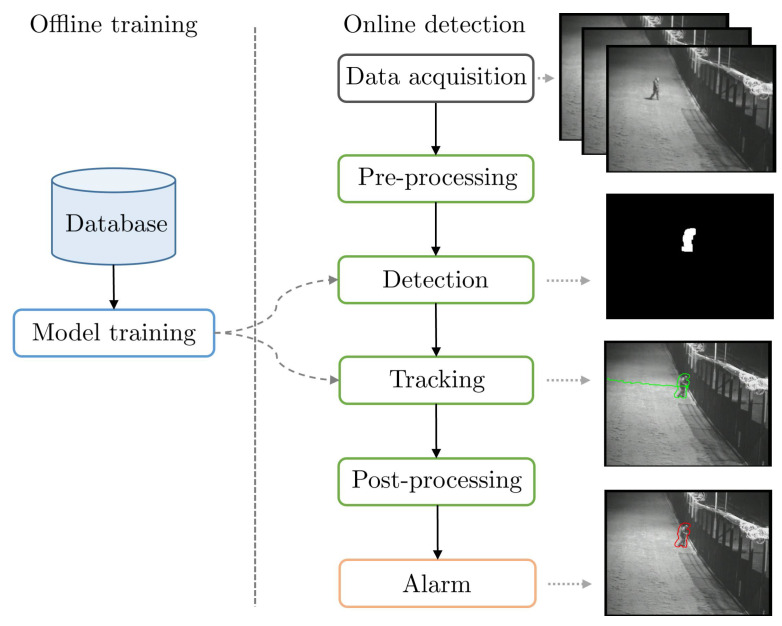
Typical pipeline of a PIDS, composed of an optional offline training (left part) and an online detection (middle part). An illustrative example of different steps in online detection is shown in right part of the figure.

Figure 3 shows the typical PIDS pipeline with various associated tasks. In order to review PID methods, we must review methods that tackled one or several of these tasks with the aim to improve the PIDS pipeline, as only a few methods tackle the complete PID task.

### 3.1. Pre-Processing

Low quality sensors and adverse environmental conditions such as snow, fog, rain, extreme sunshine, etc., may produce highly noisy video streams. We cannot directly feed this noisy data into the detection algorithm. Therefore, video enhancement is needed to remove noise and improve the visual appearance of the video.

The existing video enhancement methods can be classified into two broad categories [50,51]: spatial domain enhancement and transform domain enhancement. Spatial domain video enhancement deals directly with pixels, i.e., it makes a direct manipulation of pixels in video frames. It is conceptually simple and has a low time complexity, which favors real-time implementation but lacks robustness. Some surveys on this method can be found in [52,53,54]. In most PIDS, some standard spatial enhancement is carried out on raw frames [6,25,41], such as image resizing, image normalization, mean centering, colorspace conversion (RGB to grayscale or vice versa), histogram equalization, etc.

Transform domain video enhancement operates on the transform coefficients of the video frame, such as Fourier transform, discrete wavelet transform and discrete cosine transform [54,55]. The video quality is enhanced by manipulating the transform coefficients. This category of methods has a low computational complexity with ease of manipulating the frequency composition of the video frame. Some major examples of PIDS using these techniques are [25,41], using fast Fourier transform (FFT) on video frame patches and decreasing noise by removing very low or high frequencies from FFT.

Apart from video enhancement, some other pre-processing can be conducted depending on the PIDS. In [25], patches of 16-pixel squares in each video frame are defined and two regions (grass and fence area) are further designated for segregating the scene into an authorized/unauthorized zone. Another common pre-processing is to have a fixed spatial perimeter in each frame of the video [6,22,24,47]. This helps the PIDS to focus only on this region of the scene and to ignore activities outside this perimeter.

### 3.2. Detection

This is an important step of the pipeline as the goal of PIDS is to detect certain categories of objects that might cause an intrusion. There are two main families of detection in video: (i) detection of blobs, analyzing the pixel motion, and (ii) detection of objects, analyzing the image appearance with the localization and classification of objects.

#### 3.2.1. Motion Detection

The act of intrusion is caused by a moving object in a protected perimeter during an unauthorized time. Therefore, motion detection is essential in a PIDS. The main approaches can be classified into three categories.

##### Optical Flow

The notion of optical flow literally refers to the displacements of intensity patterns. It is an approximation of image motion defined as the projection of velocities of 3D surface points onto the imaging plane of a visual sensor [56]. Optical-flow-based methods use partial derivatives with respect to the spatial and temporal coordinates to calculate the motion between video frames. However, most optical flow methods are computationally complex, very sensitive to noise and tough to implement in real-time settings. Some surveys on optical flow approaches are [56,57].

##### Temporal Differencing

Temporal differencing uses pixel-wise differences among consecutive video frames to extract moving regions. It is adaptive to dynamic environments and has a low computational complexity. However, it can fail to extract all of the relevant pixels and can leave holes in regions. Some important studies can be found in [2,24,58,59]. The studies in [25,41] use simple inter-frame differencing followed by some morphological operations for motion detection in their PIDS.

##### Background Subtraction

Background subtraction is one of the key techniques for detecting moving objects in video. It detects moving regions by taking the difference between the current frame and the reference frame, often referred to as the ‘background model’. The detection ability depends on the adaptiveness of the background model. Some popular background subtraction methods are: running Gaussian average (RGA) [60], Gaussian mixture model (GMM) [61], kernel density estimator (KDE) [62] and visual background extractor (ViBe) [63]. Background subtraction mainly suffers from illumination changes, a dynamic background, shadows, camouflage, video noise, etc. [40]. These effects can present a background object as a false foreground moving object or vice-versa. Most comprehensive surveys on background subtraction-based methods are [19,33,40].

Concerning PIDS, Refs. [25,41] use Gaussian background modeling to discriminate people (intruder) from background. The study in [4] uses background subtraction to extract object blobs from the video frame, which are later used for tracking. The study in [22] detects moving objects by comparing a background model with an input video frame in real-time. The study in [24] detects objects using the RGA method.

#### 3.2.2. Object Detection

Object detection, i.e., object localization and classification, has been a field of intensive research, and intrusion detection is closely related to it. In fact, intruders belong to certain categories of objects, such as people, car, bike, etc., to be detected in a protected area. Even though intrusion can be caused by vehicles, animals, etc., the state of art mostly focuses on detecting people. Object detection methods can be categorized into traditional and deep-learning-based detectors [20]. Some traditional object detectors are the Viola–Jones detector [64], histograms of oriented gradients (HOG) detector [65] and deformable part-based model (DPM) [66]. With the advent of deep learning, we have achieved an excellent performance in object detection with methods such as Faster R-CNN [67] and YOLO [68]. Still human detection can be challenging, especially in scenes with an atypical human pose, such as crawling/creeping, occluded scenes [69] and scenes with low luminosity, such as during night. The study in [70] addresses the problem of detecting humans at night using a consistency–constancy bi-knowledge learning network that exploits the cross-time (day and night) scene consistency and cross-frame background constancy. In [22], a 2D CNN-based supervised classifier for human intruder detection is used. Similarly, Ref. [47] uses a pre-trained YOLO v2 network for intruder object detection.

### 3.3. Tracking

Tracking objects can be useful for a PIDS. Perimeter protection solutions may use this information to impose detection constraints. For example, leaving an area can be allowed, but not entering it. We can also think of raising an intrusion alarm only if an object is inside the area for a specified amount of time. Furthermore, an efficient tracking module can help a PIDS to not lose an object and re-initialize its timer.

A Kalman filter is applied on the texture of objects with a motion mask to build object tracks [41]. Particle filters can be used too to track intruders [22,24]. In [25], an intruder is tracked by logging positions of foreground objects over time. The study in [48] proposes a tracking algorithm based on tracklet clustering. Finally, [47] uses the simple on-line and real-time tracking (SORT) algorithm [71] for intruder tracking.

### 3.4. Joint Detection and Tracking

Since a video has two components, spatial and temporal, it is usually analyzed in two steps. The first step captures spatial patterns by using detection on each frame (see Section 3.2), whereas the second step uses tracking to apprehend temporal coherence (see Section 3.3). This approach creates the hypothesis that spatial and temporal dimensions are independent and can be processed sequentially.

Recent approaches jointly model spatial and temporal dimensions using 3D convolutions and improve results in video analysis [72,73]. Applied to PIDS, an implicit joint detection and tracking is performed by a 3D convolutional autoencoder in [6], trained in an unsupervised way.

### 3.5. Post-Processing

Missing intrusions in the site are considered a major failure for a PIDS; therefore, methods try to detect as much as possible, even at the cost of some false alarms [24]. These false alarms need to be filtered, which is why we might need some sort of post-processing. Even though this step is crucial in a PIDS, there are few publications on this topic because manufacturers prefer to keep their post-processing confidential. However, despite this, we can list several post-processing techniques.

Filtering objects of interest outside the chosen perimeter is the most common post-processing and is used in major PIDSs [22,24,47]. Sometimes, blobs are inconsistent across time, such as rain drops, and a filter can check the coherence of the blob trajectory. Detected objects can also be filtered with a minimum threshold on the blob size. For example, Ref. [24] filters all of the objects with a size less than four pixels. Since foreground objects are bigger than background ones, perspective calibration learns the dimension of object of interest as a function of its position in the scene. This allows us to filter objects with a size smaller than the expected size of the object of interest at the same position in the scene [74,75].

### 3.6. Alarm

To transform detected and tracked objects into alarms, PIDSs apply some sensibility thresholds to set the omissions–false alarms trade-off [4,22,41,47]. These thresholds are usually manually tuned during the actual deployment of the PIDS.

Moreover, some high-level rules can be applied to trigger the alarm. In [25], the alarm is triggered if the intruder shows movement towards the target for a minimum time of 2 s. In [22,47], alarms are generated as long as the intruder is inside the protection boundary, while [24] adds an extra constraint, where it must be tracked for at least three frames.

Interestingly, some publications evaluate PIDS in a pure machine learning approach, using metrics that integrate performances for all possible threshold values [6,7]. However, they do not describe a strategy to choose the thresholds, making it difficult to use them in practical cases.

### 3.7. System Deployment

The actual deployment of the system is realized in three stages: an optional offline model training, then an online initialization of the system, and, finally, online execution.

#### 3.7.1. Model Training

Some PIDSs require offline training on part of the dataset for their detection or tracking steps [6,22,47]. This training can be supervised, requiring labeled videos (tagging intrusion frames or events); or, it can be unsupervised, under the assumption that there are no annotated data in the dataset. In [4], a classifier model is trained as a multiple instance learning problem by employing image-based features to distinguish intruder objects from moving vegetation and other distractions. The studies in [22,47] use supervised object detectors for intruder detection. The study in [6] learns an unsupervised deep autoencoder on training videos having no intrusions in order to detect intrusions in testing videos.

#### 3.7.2. System Initialization

During real-life deployment, most of the PIDS must be initialized for several seconds in order to set the system’s internal state of the system aligned with the new scene in order to protect [24,25]; for example, the mean and standard deviation of a GMM. This online initialization must not be confused with the offline model training. Moreover, a PIDS can have sensibility thresholds, which are manually tuned by the installer during the deployment.

#### 3.7.3. System Execution

The last stage is the online execution of the PIDS. It includes all of the steps of the pipeline as illustrated in the right part of Figure 3. To provide a reliable protection, most of the PIDSs work between 5 and 25 FPS [6,24,25].

Table 2 summarizes major PIDSs with various methods used in different steps of the pipeline and the availability of the source code. We can observe that most systems use the visual camera for data acquisition. Only one system uses the thermal camera and just one system is multi-camera-based. Background modeling is used in most traditional systems for detection, whereas deep-learning-based models use 2DCNN, a YOLO detector and autoencoders. Regarding tracking, the Kalman filter, particle filter, tracklet-based tracking and SORT are used. For alarms, most systems have their own rules depending on the method. Three systems use supervised training, one system uses unsupervised training and the rest do not include a training step.

## 4. Definition of PID Task for Video Surveillance

The task of perimeter intrusion detection (PID) has been defined in various ways in the state-of-the-art. In [4], it is defined as a monitoring system that identifies the presence of humans or devices in a pre-defined field-of-view. In [22], the PIDS is defined as a system that detects physical intrusions on a site having a protective barrier to isolate it from outside. In [23], it is described as a system that detects the movements of intruders attempting to breach a security wall or region and alert security. However, all of these definitions lack clarity and formalization; for example, the following questions need to be addressed: “what are intruders?”, “does moving intruder cause intrusion?” and “is a protective area necessary?”. To answer all of these questions, we mathematically define a PIDS. Before defining a PIDS, we must define what an intrusion is.

### 4.1. Intrusion in the Video

To properly define an intrusion, we need to define an object in the video.

#### 4.1.1. Object in the Video

**Figure 4:**
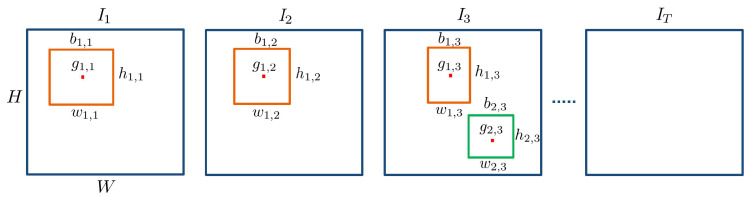
Video with *T* frames of height *H*, width *W* and channels *D* = 1. Two objects, shown in orange and green bounding boxes, are defined as $o_{1}=\left(\left\{b_{1,1},b_{1,2},b_{1,3}\right\},c_{1}\right)$ and $o_{2}=\left(\left\{b_{2,3}\right\},c_{2}\right)$, where $c_{1},c_{2}\in C$ are the object classes. Here, $\left\{b_{1,1},b_{1,2},b_{1,3}\right\}$ are bounding boxes of object 1 on first three frames and $\left\{b_{2,3}\right\}$ represents bounding box of object 2 at frame 3.

We define a video V acquired for *T* frames during the interval T=[1,T] as:

(1) $$V={\left\{I_{t}\in R^{H\times W\times D}\right\}}_{t\in T}\;,$$

where $I_{t}$ denotes the frame at the time instant *t*, with height *H*, width *W* and number of channels *D*. To define an object in the video, we must first specify the object definition at frame-level. An object in a frame or image is defined with a spatial specification and a class that distinguishes one family of objects from another (such as humans, animals or cars). The spatial specification can be either on pixel-level by allocating each pixel to an object or background, or on area-level by encapsulating the object in a bounding box. We choose the bounding box as it has been used in the literature extensively [20]. It should be noted that the choice of spatial specification cannot have an impact on the intrusion definition. Thus, we define an object at frame-level with a class and a bounding box. To define an object in the video, we take into account all of the frames where it is present. Therefore, an object $o_{i}$ in the video is defined as:

(2) $$o_{i}=\left({\left\{b_{i,t}\right\}}_{t\in T},c_{i}\in C\right)\;,$$

where $c_{i}$ is the class of the object from the set of object classes C, and $b_{i,t}$ is its bounding box at time instant *t*, which is defined as:

(3) $$b_{i,t}=\left\{g_{i,t},w_{i,t},h_{i,t}\right\}\;,$$

where $g_{i,t}$, $w_{i,t}$ and $h_{i,t}$ are the center, width and height of the bounding box, respectively. The center is defined by its coordinates as $g_{i,t}=\left(x_{i,t},y_{i,t}\right)\in I_{t}$. Note that, instead of the bounding box center, it is possible to choose other points, such as the bounding box bottom, as reference. We illustrate these definitions in Figure 4.

#### 4.1.2. Intrusion Event and Intrusion Interval

For the protection of a site, the user must define parameters that qualify objects as non-authorized (na), i.e., intruders.

- $S_{na}\subseteq R^{H\times W}$ : the subset of the frame/image, defining the surface to protect.
- $T_{na}\subseteq T$ : the time interval during which the surface must be protected (e.g., protection during night).
- $C_{na}\subseteq C$ : the set of non-authorized classes, such as person, car, truck, etc. These classes of objects are considered as possible intruders and can be different according to site and client demands.

Since $C_{na}$ is a non-finite set (it is impossible to make the exhaustive list of non-authorized objects, exposing the system to omissions), it is easier to ask the user to explicitly define the short list of authorized objects $C_{a}$ (such as small animals), which leads to $C_{na}=C\backslash C_{a}$.

**Figure 5:**
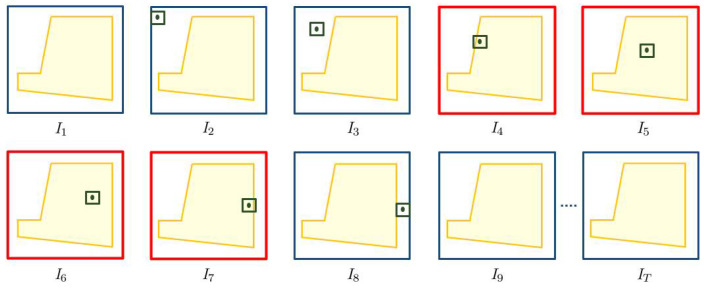
Illustration of an intrusion event caused by a single object. Video with *T* frames, where $S_{na}$ is shown with yellow surface and object with a green bounding box plus center. The object causes an intrusion event for four frames from frame $I_{4}$ to $I_{7}$, colored in red.

An object causes an intrusion event if it belongs to a non-authorized class and is moving in a protected area during a prohibited time interval. We define the intrusion event caused by an object $o_{i}$ as:

$$\begin{array}{cc} \mathrm{IE}\left(o_{i}\right)=\{\; & I_{t}\;\;s.t.\;\;c_{i}\in C_{na}\;\mathrm{and}\;t\in T_{na}\;\mathrm{and}\;∥\mathrm{grad}\;{\vec{g}}_{i,t}∥>0\;\mathrm{and}\;g_{i,t}\in S_{na}{\;\}}_{t\in T}\;, \end{array}$$

where $∥\mathrm{grad}\;{\vec{g}}_{i,t}∥$ is the gradient of object $o_{i}$ at instant *t* and it being non-zero signifies that the object is in motion. Thus, the intrusion event caused by object $o_{i}$ is a collection of all of the frames $I_{t}$ such that $t\in T_{na}$, object class $c_{i}\in C_{na}$, the gradient is non-zero and the bounding box center lies in the protected area. Figure 5 illustrates the intrusion event caused by an object. The surface to protect $S_{na}$ is depicted with a pink trapezoid in each frame, and we assume that we want to protect during the entire video. One object is present in the video and it is shown with a rectangular bounding box plus a center. The object is in motion from the second frame to the eighth frame. While in motion, the object’s center lies in $S_{na}$ from the fourth frame until the seventh frame, causing an intrusion event. Thus, this object triggers an intrusion event for four frames.

Since a video can have more than one object causing intrusion events, we define the intrusion event of the whole video containing *j* objects as:

(4) $$\mathrm{IE}\left(V\right)=\overset{j}{\underset{i=1}{⋃}}\mathrm{IE}\left(o_{i}\right)\;.$$

**Figure 6:**
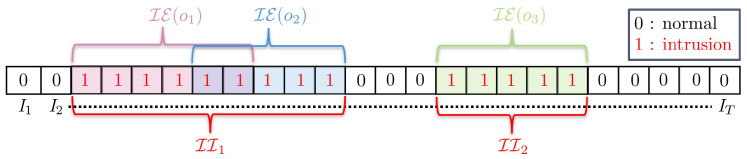
Illustration of intrusion event of the video and intrusion intervals. Objects $o_{1}$, $o_{2}$ and $o_{3}$ cause intrusion events $\mathrm{IE}(o_{1})$, $\mathrm{IE}(o_{2})$ and $\mathrm{IE}(o_{3})$, marked with value 1. IE(V) is collection of all of the frames with value 1. Two intrusion intervals ${\mathrm{II}}_{1}$ and ${\mathrm{II}}_{2}$ are shown in red intervals.

Figure 6 shows three intrusion events caused by three objects in the video. We can observe that intrusion events of object 1 and 2 overlap for two frames, meaning that, for those two frames, there were two objects causing intrusion events simultaneously. The intrusion event of this video is a collection of all of the intrusion frames, marked by 1 in the figure. In the context of video surveillance, we are concerned with whether there is an intrusion event or not, regardless of whether one object or many objects are causing it. Therefore, we are interested in an interval of a contiguous sequence of intrusion frames. We term this as an intrusion interval, and the task of intrusion detection is focused on detecting them. Formally, an intrusion interval II⊆IE(V) is defined on a closed interval as:

$$\begin{array}{cc} \mathrm{II}=\{\; & I_{t}\in \mathrm{IE}\left(V\right)\;\mathrm{with}\;t\in \left[t_{start},t_{end}\right]\;s.t.\;I_{t_{start}-1}\notin \mathrm{IE}\left(V\right)\;\mathrm{and}\;I_{t_{end}+1}\notin \mathrm{IE}\left(V\right)\;\}\;, \end{array}$$

where $t_{start}$ and $t_{end}$ denote the first and last frames of an intrusion interval. In other words, an intrusion interval is a contiguous sequence of frames of maximal size derived from IE(V). Figure 6 depicts two intrusion intervals of the video.

### 4.2. PIDS

Given a precise definition of intrusion, we can now define a perimeter intrusion detection system (PIDS). Given a video V and intrusion parameters $\left(S_{na},T_{na},C_{na}\right)$, the prediction of a PIDS can be defined as:

(5) $$P\left(V,S_{na},T_{na},C_{na}\right)={\left\{{\hat{p}}_{t}\in \left\{0,1\right\}\right\}}_{t\in T_{na}}\;,$$

where ${\hat{p}}_{t}$ is a binary prediction for each frame *t* of video V for time $T_{na}$, with 1 denoting a frame predicted as an intrusion, and 0 otherwise. Therefore, a PIDS classifies each frame into an intrusion frame or otherwise. This type of output is useful when we want to evaluate a PIDS at frame-level [6]. In a real-life surveillance system, the system sends an alarm signal to surveillance personnel as soon as there is a transition from a normal to intrusion state [26]. The output of the PIDS can be derived from P as follows:

$$\begin{array}{cc} A(P)=\{\; & {\hat{p}}_{t}\in P\;\;s.t.\;\;{\hat{p}}_{t-1}\in P\;\mathrm{with}\;{\hat{p}}_{t-1}=0\;\mathrm{and}\;{\hat{p}}_{t}{=1\;\}}_{t\in T_{na}}\;. \end{array}$$

This is a set of intrusion alarms created by the system, marked by the rising edge, i.e., the transition of the system state from non-alarm to alarm. These alarms alert the surveillance personnel about a suspicious activity. For each alarm, a mini-clip is sent containing some frames before the alarm and some frames after the alarm. The surveillance personnel visually analyze this mini-clip and decide whether it is an actual intrusion activity or a false alarm. Therefore, we need an evaluation scheme that takes into account this real-life scenario.

## 5. Evaluation Protocols

Given a video or set of videos, the PIDS detects intrusions. To evaluate the performance, we need to compare the PIDS output with ground truth annotations. The manner in which this evaluation is carried out impacts the final score metric. The following subsections present different evaluation protocols.

### 5.1. Frame-Level Evaluation

As the name suggests, in this type of evaluation, we are interested in checking whether each frame of the video is correctly classified as intrusion/normal or not. For a video V and given intrusion parameters ($S_{na},T_{na},C_{na}$), the frame-level ground truth is defined as:

$$\begin{array}{c} G(V,S_{na},T_{na},C_{na})={\left\{\;p_{t}\in \left\{0,1\right\}\;s.t.\;p_{t}=1\;\mathrm{if}\;p_{t}\in \mathrm{IE}\left(V\right)\;\right\}}_{t\in T_{na}}\;, \end{array}$$

where $p_{t}$ is the ground truth label for each frame *t* of the video V at time $T_{na}$; value 1 denotes an intrusion class, and 0 otherwise.

Given ground truth G and prediction P (see Equation (5)), the frame-level intrusion evaluation is simply the binary classification evaluation of each frame of the video [76]. We can calculate elements of the confusion matrix, i.e., the true positive (TP), false negative (FN), false positive (FP) and true negative (TN), with intrusion as the objective class [6]. We can then evaluate the performance of the PIDS depending on the choice of metric, such as the precision, recall, $F_{1}$ score, etc., as defined in Section 5.4.

In this type of evaluation, each frame contributes equally to the overall score. Thus, it can provide the same overall score for an algorithm that gives us multiple omissions of intrusion events versus an algorithm that gives us an omission of some intrusion frames in multiple intrusion events. This is an undesirable evaluation in the case of intrusion detection because we cannot afford to have omissions of intrusion events. In reality, we are more interested in knowing if the system is able to classify the intrusion events correctly as a whole. This demands an event-level evaluation. In other words, we want to detect all intrusion intervals (IIs) from the video. More specifically, we are interested in evaluating whether the beginning of these intrusion intervals are detected correctly. This is because, if an intrusion event is detected too late, then that detection is not very useful. The idea is to detect each intrusion interval as soon as it occurs and, thus, we need an evaluation scheme that takes this into account.

### 5.2. i-LIDS Evaluation

For evaluating on the i-LIDS dataset, their user guide provides an evaluation procedure [26]. It focus on evaluating intrusion at event-level rather than frame-level. To be precise, an intrusion is considered correctly detected if there is at least one system alarm within 10 s from the start of the intrusion event. For an II of the video and alarms A(P), the rules of the i-LIDS evaluation protocol are as follows:

1. TP: if there is at least one alarm within 10 s from the beginning of the II. If there are multiple alarms candidates, the first one is taken and the rest are ignored.
2. FN: if there is no alarm within 10 s from the beginning of the II.
3. FP: if there is an alarm but not within 10 s from the beginning of the II. If there are consecutive FPs within a 5-s gap among them, only the first one is considered and the rest are ignored.

**Figure 7:**
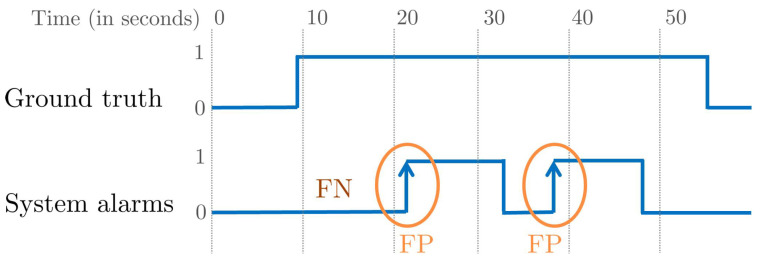
Illustration of i-LIDS evaluation protocol, highlighting its drawback on an intrusion example starting at 9th second. Since no alarm has been raised in the first 10 s of the intrusion, a FN is counted. Following alarms, at 22nd and 37th second, are marked as FP because they do not occur within 10 s from the beginning of the intrusion.

Apart from these, one rule is i-LIDS-dataset-specific: all IIs and alarms that start within 5 min from the beginning of the video are ignored. This means that they wanted to give a preparation time to the system. This evaluation scheme is not generic and has several drawbacks, as illustrated in Figure 7. It penalizes an alarm as an FP after 10 s from the beginning of an II without taking into account the duration of intrusion. If the II has a long duration (such as an hour) and we have an alarm at the 11th second, it is not ideal to mark it as an FP. From a practical point of view, the surveillance personnel will receive a mini-clip as soon as the alarm is triggered and, if the intrusion is present, then it is not sensible to mark this as an FP. Instead, this alarm should be ignored as it is not detected within 10 s. Similarly, each alarm after 10 s but within II is considered as an FP, and this strongly penalizes the system precision. Instead, these extra alarms should be counted without assigning them as an FP.

### 5.3. Edge-Level Evaluation

**Figure 8:**
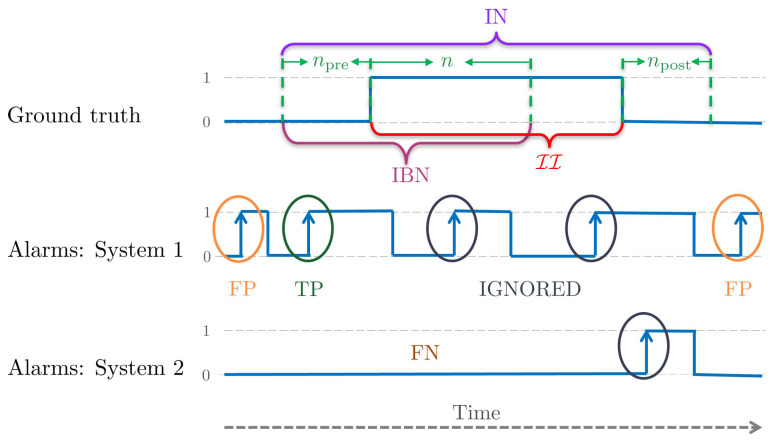
The top subfigure is the illustration of the definitions of edge-level evaluation terms on ground truth, with time in abscissa and non-intrusion (0) and intrusion (1) class for each frame of the video in ordinate. The next two subfigures represent examples of alarms and possible outcomes (TP, FP, FN) for two different PIDSs evaluated by the edge-level protocol.

To appropriately evaluate a PIDS while considering the real-world aspects, we propose a new evaluation protocol. An intrusion event begins with a transition from a non-intrusion to intrusion state, i.e., we have a rising edge as shown in Figure 8. Similarly, an intrusion event stops by a reverse transition, i.e., a falling edge. We are interested in detecting intrusion within a few frames from the rising edge. Since we focus on this rising edge, we call this the edge-level evaluation. In other words, we emphasize detecting the beginning of intrusion intervals. We first define the following terms from an intrusion interval of the video (see Figure 8).

The intrusion interval neighborhood IN is an expanded interval defined by $n_{\mathrm{pre}}$ frames before and $n_{\mathrm{post}}$ frames after the II:

$$\begin{array}{cc} \mathrm{IN}(\mathrm{II} & ,n_{\mathrm{pre}},n_{\mathrm{post}})=\left[t_{\mathrm{pre}},t_{\mathrm{post}}\right]\;s.t.\;\;t_{\mathrm{pre}}=t_{\mathrm{start}}\left(\mathrm{II}\right)-n_{\mathrm{pre}}\;\mathrm{and}\;t_{\mathrm{post}}=t_{\mathrm{end}}\left(\mathrm{II}\right)+n_{\mathrm{post}}\;. \end{array}$$

These $n_{\mathrm{pre}}$ and $n_{\mathrm{post}}$ frames are in the range of one to five (less than 1/5 s for a video at 25 FPS) and are added in order to take into account the error of annotation. This error is due to the fact that it is difficult to mark the exact frame at which the intrusion starts or ends. This tolerance further permits not strictly penalizing the system when an intrusion event is detected a few frames before the actual event or when the system detects a few more intrusion frames after the actual event is finished. These cases arise often when the intrusion object is in the scene but not inside the surface to protect. Therefore, IN is an interval where the actual intrusion activity takes place, and an alarm given by a PIDS in this interval can be counted as either TP or ignored. An alarm given outside IN must be a false alarm and should be counted as an FP.

The intrusion beginning neighborhood IBN is an interval comprising $n_{\mathrm{pre}}$ frames before and *n* frames from the beginning of II:

$$\begin{array}{c} \mathrm{IBN}(\mathrm{II},n_{\mathrm{pre}},n)=\left[t_{\mathrm{pre}},t_{n}\right]\;s.t.\;\;t_{\mathrm{pre}}=t_{\mathrm{start}}\left(\mathrm{II}\right)-n_{\mathrm{pre}}\;\mathrm{and}\;t_{n}=t_{\mathrm{start}}\left(\mathrm{II}\right)+n\;. \end{array}$$

This interval signifies the importance of the initial frames of an II, where an intruder has just entered the protected area, and it is in this interval where we ideally want the PIDS to raise an alarm. An alarm raised in IBN must be a TP.

For an II and alarms A(P), the possible outcomes at edge-level are defined as (see Figure 8):

1. TP: if there is at least one alarm in IBN. For multiple alarms in IBN, only the first one is considered, and the rest are ignored.
2. FN: if there is no alarm in IBN.
3. FP: if an alarm is outside IN. Each alarm outside of IN is counted as an FP.

In this evaluation scheme, alarms lying outside IBN but inside IN are ignored. This means that we neither adversely penalize these alarms as an FP nor count them as a TP. In event-level evaluation, whether i-LIDS or this scheme, we do not define a true negative (TN). A TN is when a normal (non-intrusion) event is detected as such; in other words, how well we are classifying a normal event as normal. However, this is not the aim of intrusion detection; indeed, it is the opposite. Furthermore, the calculation of TN is ambiguous. We cannot generalize what length of the non-intrusion video should be considered as a TN. For example, a non-intrusion video clip of 5 min cannot be considered as similar to a non-intrusion video clip of 5 days.

These rules are for individual IIs, but how we deal with scenarios where the intrusion neighborhoods are so close that they intersect one another is another matter. If INs of two or more II intersect one another, then we merge them into a single IN. The new IN consists of $n_{\mathrm{pre}}$ frames of the first II and $n_{\mathrm{post}}$ frames of the last II, and all of the frames in between are merged as an II. Algorithm 1 summarizes the protocol to evaluate a video at edge-level.
**Algorithm 1:** Edge-Level Evaluation of a PIDS
1 Initialize variables *n*, $n_{\mathrm{pre}}$ and $n_{\mathrm{post}}$.
2 Calculate IN for all IIs of the video.
3 If two or more INs intersect, merge them into a single expanded IN.
4 Calculate intrusion beginning neighbourhood IBN for each II.
5 Obtain alarms A(P) from the PIDS.
6 Calculate TP, FN and FP.
7 Calculate precision, recall and other metrics.


### 5.4. Metrics

Since a PIDS has a binary classification task to classify a frame or event as an intrusion (positive) or not (negative), we can naturally apply common metrics. The following metrics are primarily suitable for a PIDS and have been widely used in the literature.

#### 5.4.1. Precision and Recall

The precision is the percentage of correctly predicted intrusions out of the total predicted alarms, as defined in the left part of Equation (6). It is particularly useful when we want to measure how false alarms are affecting the system. A high value of precision denotes that we have very low false alarms. Clients usually demand a certain minimum precision from the system.

(6) $$\mathrm{Precision}=\frac{\mathrm{TP}}{\mathrm{TP}+\mathrm{FP}}\;\;\;\;\;\;\;\;\;\;\;\;\;\;\mathrm{Recall}=\frac{\mathrm{TP}}{\mathrm{TP}+\mathrm{FN}}\;.$$

The recall is the percentage of correctly predicted intrusions out of the total intrusions, as defined in the right part of Equation (6). It is useful when the cost of the false negative is high, i.e., when we cannot afford to have an omission of intrusion. Usually, the clients prefer to have the minimum omissions possible. This means that we need to ideally maximize recall.

#### 5.4.2. $F_{\beta }$ Score

To take into account both omissions (FN) and false alarms (FP), we need a way to combine precision and recall. The $F_{\beta }$ score combines them with the β parameter as the bias to give more or less importance to recall or precision.

(7) $$F_{\beta }=\frac{(1+\beta^{2})\times \mathrm{Precision}\times \mathrm{Recall}}{(\beta^{2}\times \mathrm{Precision})+\mathrm{Recall}}\;.$$

The most common values used for β are 0.5, 1 and 2. With β = 1, we obtain the $F_{1}$ score. The $F_{1}$ score is the harmonic mean of precision and recall and it is widely used [4,48]. The choice of the value of β depends on the client needs. For the i-LIDS dataset, they propose two system roles with different bias values [26]. The roles are called ‘Operational Alert’ and ‘Event Recording’, with β as 0.81 and 0.87, respectively (In [26], they use α instead of β. For equivalency, $\alpha =\beta^{2}$). The former role is designed for real-time intrusion detection and, therefore, has a lower β value to give more importance to precision, as false alarms are essential here. The latter role is for non-real-time systems, where videos are recorded and analyzed on an offline basis. It has a higher β value, as we cannot afford omissions in this case. Most PID systems [24,25,41] use this $F_{\beta }$ metric for evaluating on the i-LIDS dataset.

#### 5.4.3. Other Metrics

The metrics listed above are threshold-based, i.e., they depend on a single chosen threshold of the classifier. Therefore, systems tend to choose a threshold to maximize the final score, e.g., the study in [25] chooses a high detection threshold to eliminate false alarms, as the metric used is $F_{\beta }$ with β = 0.81, which favors precision. Thus, the results from these metrics tend to overfit the dataset, the choice of metric hyperparameters such as β, etc.

Alternatively, PIDS can be compared over a range of all possible thresholds, avoiding bias evaluation by the choice of a given threshold. For this, we can either build the receiver operating characteristic (ROC) curve or the precision–recall (PR) curve [76,77]. The area under the curve (AUC) of ROC or PR curves gives an overall score between 0 and 1, where 1 is the best possible score. Since intrusions are rare events in videos, it is frequent to have PID datasets with a very low number of abnormal (intrusion) frames compared to the normal (non-intrusion) frames. When classes are highly imbalanced, such as in PID, AUC-PR (AUPR) must be used to compare methods [6,77], rather than AUC-ROC (AUROC), which gives inconclusive results. Then, when deploying the PIDS in a real site, the value of the threshold must be defined and the results can be finally reported with precision, recall and $F_{\beta }$ scores.

## 6. Comparison of Evaluation Protocols for PIDS

In this section, we will compare the different evaluation protocols to evaluate the existing PID systems.

### 6.1. Experimental Setup

#### 6.1.1. Data

We used the i-LIDS sterile zone dataset with the two cameras views as described in Table 1. We also considered the three intrusion distances (close, middle and far) provided with the dataset. We resampled the dataset to 5 FPS and manually verified and adjusted annotations.

#### 6.1.2. Methods

We compared the methods for which the source code is available (see Table 2): [47], used as is, and [6], completed with output alarm in order to be comparable.

The first method [47] follows the typical PIDS pipeline (see Figure 3), along with some deep-learning-based components. Given a video, frames are extracted and the user is asked to draw a perimeter in the frame for protection. Then, the user is asked to choose a potential intruder from pre-defined classes, such as person, car, etc. The system detects the intruder object with bounding box using the pre-trained YOLO v2 network [68] and a detection threshold of 0.25, only for class human. If the detected intruder object is inside the pre-defined perimeter, then it is considered as an intrusion. Finally, the object is tracked using simple online and real-time tracking (SORT) algorithm [71]. For testing it in i-LIDS, we first drew a protection perimeter following the fences for each view. We then chose person as an intruder class. Then, frames of each video are fed into the system and we obtained a binary intrusion/non-intrusion class for each frame.

The second method [6] is an unsupervised deep-learning-based approach where a 3D convolutional autoencoder is trained on normal videos (without intrusion). While testing, intrusion detection is conducted by marking video frames with high reconstruction error. Since model requires grayscale video frames as input, i-LIDS dataset is converted in grayscale before utilization. For each video frame, a pre-processing is performed with histogram equalization. UpSampling architecture is trained on frames of original i-LIDS dimensions (720 × 576), with same number of layers, but with (32,16) and (16,32) filters in encoder and decoder, respectively. This neural architecture contains 60,889 trainable parameters. Using only non-intrusion videos, model was trained on view 1 of i-LIDS training set and tested on view 1 of i-LIDS test set (same procedure for view 2). Test uses the same protection perimeter as defined for first method above. Finally, Ref. [6] presents their results in terms of AUPR (see Section 5.4), which is threshold-independent. In real-life PIDS, thresholds and other parameters must be fixed in order to decide whether the current frame of video has intrusion or not. Therefore, we added an online z-score threshold [78], applied on window-level reconstruction error score and updated after each frame, allowing us to compare results with [47].

#### 6.1.3. Evaluation Protocols

We used the three evaluation protocols studied in Section 5: frame-level (FL), i-LIDS and edge-level (EL) evaluations. For edge-level evaluation, we set the tolerance variables $n_{\mathrm{pre}}$ and $n_{\mathrm{post}}$ to 3, i.e., less than 1 s of tolerance. For variable *n*, the following values were chosen: 5, 10 and 50. This signifies that we tested the methods to detect within 1, 2 and 10 s from the beginning of intrusion. The parameters for 1 and 2 s were selected to satisfy the demands of a real-time PIDS, whereas the parameter for 10 s was chosen in order to be comparable with i-LIDS evaluation protocol. For each evaluation protocol, we present the results in terms of precision, recall and $F_{1}$ score as defined in Section 5.4.

### 6.2. Results

We will first present the overall results on view 1, then results at different distances on view 1, and finally conclude with results on view 2. To avoid repetition in this section, we will refer to the methods of Nayak et al. [47] and Lohani et al. [6], with online z-scores as Nayak2019 and Lohani2021+zscore, respectively.

#### 6.2.1. Overall Results for View 1

**Figure 9:**
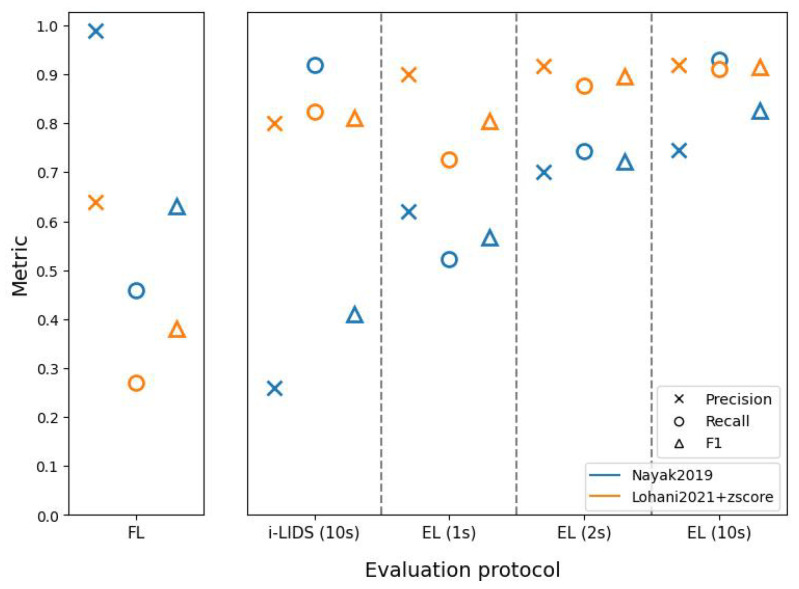
The results on view 1 of i-LIDS test set for two methods: [47] (in blue) and [6] with z-score (in orange). The abscissa represents three evaluation protocols: frame-level (FL) (on left subfigure), i-LIDS and edge-level (EL) (on right subfigure). i-LIDS is evaluated by default for 10 s whereas, for EL, we show results for 1, 2 and 10 s. The ordinate represents values of three metrics: precision, recall and $F_{1}$ score.

Figure 9 shows the results of two methods on view 1 of the i-LIDS test dataset through different evaluation protocols (see Appendix B, Table A1 for tabular results). To better understand these results, we must comprehend how these two methods predict intrusions.

**Figure 10:**
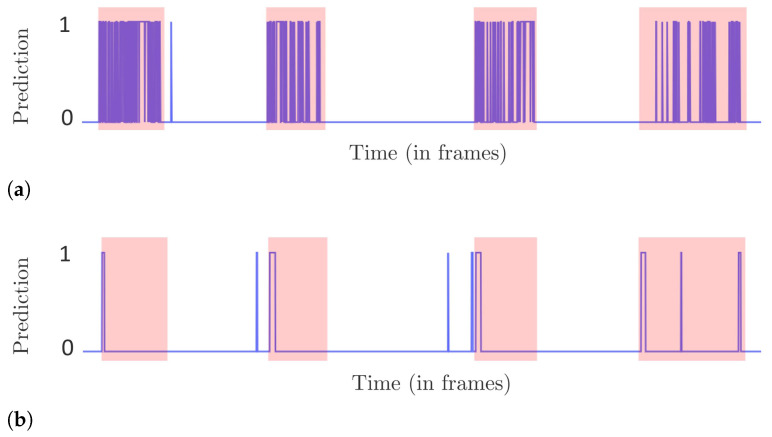
Example of per-frame predictions of two methods on a portion of video taken from i-LIDS test dataset. The intrusion intervals are shown in light red strips, the abscissa represents frames and ordinate shows prediction, where 1 signifies intrusion and 0 otherwise. (**a**) Nayak et al. [47]. (**b**) Lohani et al. [6] with z-score.

Figure 10 shows predictions using these methods on a portion of the video taken from the i-LIDS test dataset. It can be observed that Nayak2019 has a higher number of correct frame predictions in intrusion intervals compared to Lohani2021+zscore. We can also see that Nayak2019 has a smaller number of false detections than Lohani2021+zscore. These two observations have a direct consequence on the frame-level results as shown in the left subfigure of Figure 9. We see that Nayak2019 has a higher recall and precision than Lohani2021+zscore.

Since an intrusion alarm is raised as soon as a system changes its state from 0 to 1, it is important to take these state changes into account. For the i-LIDS evaluation protocol, if there is at least one alarm in the first 10 s of intrusion, it is counted as a TP. However, alarms after 10 s are counted as an FP, as already explained in Section 5.2. It can be observed that Nayak2019 has a high number of intrusion alarms from the beginning to end of each intrusion interval. These alarms at the end are considered as false detections. This explains the poor precision score of Nayak2019 in the i-LIDS evaluation protocol as shown in Figure 9. Since this method rarely misses producing alarms within the first 10 s, we have a very low number of FNs, and this is depicted with a high recall score in Figure 9. Lohani2021+zscore has a lower number of alarms, and most of them occur at the beginning of intrusions as shown in Figure 10. This leads to a smaller FP due to fewer late predictions, and we have a good precision value as seen in Figure 9. This method also has few omissions within the first 10 s; therefore, we observe a good recall value in terms of i-LIDS evaluation.

In the edge-level evaluation protocol, if an alarm is not raised within the first *n* frames or seconds of an intrusion, then it is considered as an omission or false negative. Since it is a difficult task to raise the alarm in 1st second of an intrusion, EL (1 s) has a low recall for both methods (Figure 9). As we evaluate for 2 and 10 s, both methods have more time to raise an alarm and we observe an increase in recall. Nayak2019 has a lower recall than Lohani2021+zscore in EL (1 s) and EL (2 s) because the former sometimes raises an alarm too late as shown in the rightmost intrusion interval of Figure 10. Regarding precision, here, the excessive late alarms of Nayak2019 are not penalized as FPs. Therefore, in contrast to i-LIDS evaluation, we observe a better precision value for Nayak2019 in edge-level evaluation. Lohani2021+zscore raises fewer false alarms than Nayak2019 and, thus, has a better precision in edge-level evaluation.

Overall, we observe that Nayak2019 fluctuates frequently between non-intrusion and intrusion prediction, thus producing a high number of alarms. This is because it is focused on human detection and it often loses track of the human from one frame to another, and then re-detects it. Furthermore, it has very few predictions outside intrusions. That is why we have a good precision and recall value at frame-level. However, as we go to i-LIDS and edge-level, we observe that we have a low precision value. This is because, from this high number of alarms, only few are relevant, i.e., the ones at the beginning of intrusions. Other alarms are either treated as false alarms or discarded depending on the protocol. The aim of intrusion detection is to detect the intrusion as soon as possible and not just to classify each frame; therefore, it is important to use the right protocol that emphasizes it. For Lohani2021+zscore, we observed that it has a lower number of alarms. It focuses more on the beginning of intrusion events and, due to the z-score, it quickly adapts itself, thus producing intrusion predictions for only a few frames. This is why we have a poorer frame-level score. However, in both i-LIDS and at edge-level, we have a good score because this method raises the alarm as soon as the intrusion occurs, and this is what we expect from a PIDS.

To understand where these methods struggle, we now present results based on the intruder distance from cameras.

#### 6.2.2. Results for Intrusions at Different Distances from Camera for View 1

**Figure 11:**
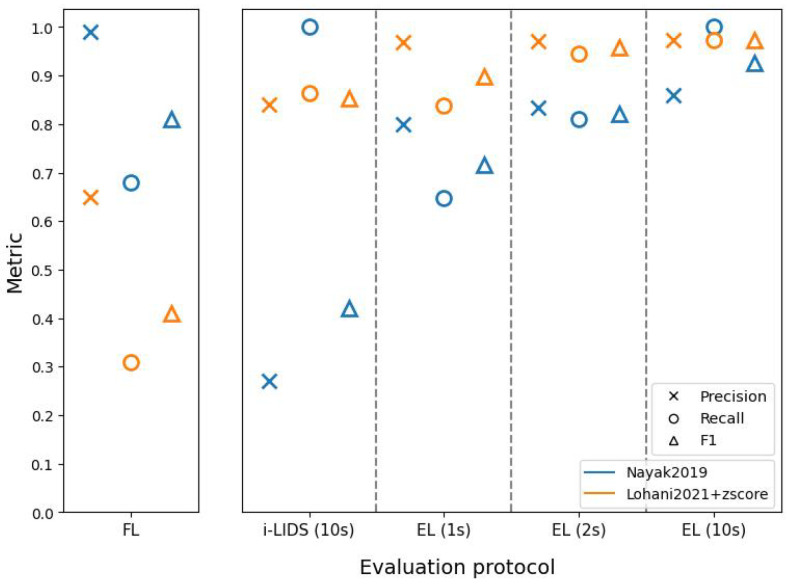
The results of two methods: Nayak et al. [47] (in blue) and Lohani et al. [6] with z-score (in orange), tested on view 1 of i-LIDS test set with intruder at close distance from camera. The abscissa represents three evaluation protocols: frame-level (FL) (on left subfigure), i-LIDS and edge-level (EL) (on right subfigure). i-LIDS is evaluated by default for 10 s whereas, for EL, we show results for 1, 2 and 10 s. The ordinate represents values of three metrics: precision, recall and $F_{1}$ score.

**Figure 12:**
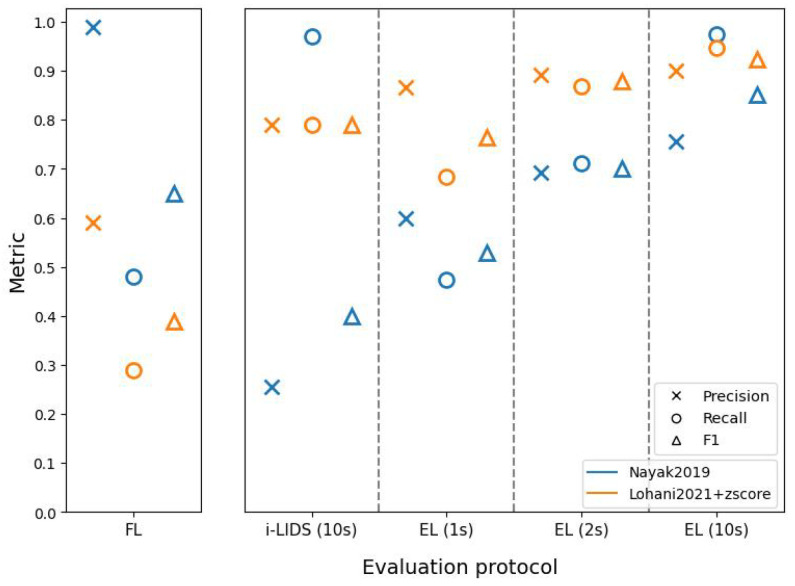
The results of two methods: Nayak et al. [47] (in blue) and Lohani et al. [6] with z-score (in orange), tested on view 1 of i-LIDS test set with intruder at mid distance from camera. The abscissa represents three evaluation protocols: frame-level (FL) (on left subfigure), i-LIDS and edge-level (EL) (on right subfigure). i-LIDS is evaluated by default for 10 s whereas, for EL, we show results for 1, 2 and 10 s. The ordinate represents values of three metrics: precision, recall and $F_{1}$ score.

**Figure 13:**
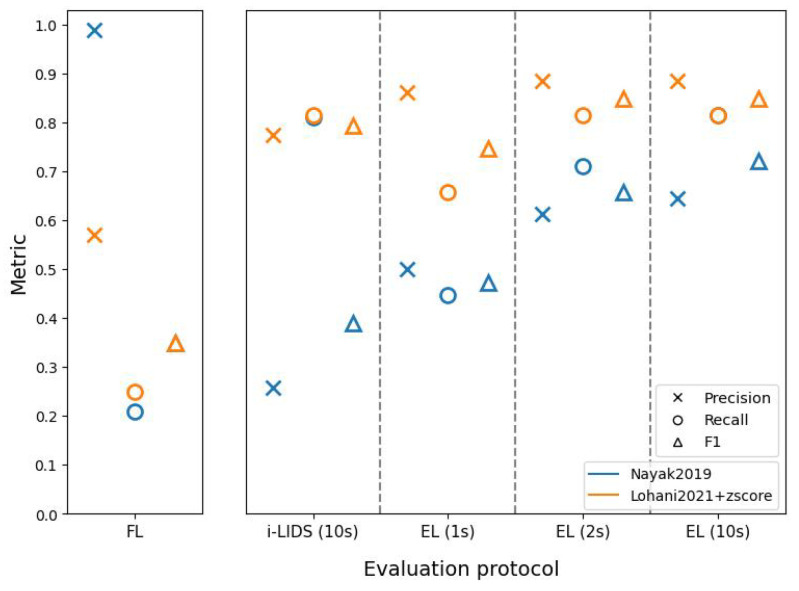
The results of two methods: Nayak et al. [47] (in blue) and Lohani et al. [6] with z-score (in orange), tested on view 1 of i-LIDS test set with intruder at far distance from camera. The abscissa represents three evaluation protocols: frame-level (FL) (on left subfigure), i-LIDS and edge-level (EL) (on right subfigure). i-LIDS is evaluated by default for 10 s whereas, for EL, we show results for 1, 2 and 10 s. The ordinate represents values of three metrics: precision, recall and $F_{1}$ score.

Figure 11, Figure 12 and Figure 13 present results of the two methods at close, middle and far distances from the camera using different evaluation protocols on the i-LIDS test dataset for view 1. We observe that, regardless of protocols or methods, we have the best performances when the intruder is close to the camera and the worst performances when the intruder is far.

We also observe in these three figures that the frame-level evaluation follows the same trend as the overall results and does not add significant information to draw conclusions. Therefore, we focus on i-LIDS and edge-level results for interpretation.

Edge-level results of Nayak2019 at three distances show that the recall increases rapidly as we move from 1 s towards 10 s. Visual inspecting revealed that these events consisted of intrusions where the intruder was not standing straight, i.e., the intruder was crawling, body dragging, log rolling, etc. Given 1 or 2 s, Nayak2019 was not able to detect these intrusions. Nayak2019 uses YOLO trained on VOC2012 [79], a dataset containing only standing humans. Since it can only detect what it has learned on the dataset, YOLO fails to detect intrusions performed by non-standing humans. However, eventually, these intruders stood up and, if it happened within the first 10 s, then they were detected. We can see that these recall scores decrease with an increased distance from the camera, the reason being a smaller intruder object size, along with activities such as crawling. The precision scores remain almost similar with time. This is because the false positives remain detected regardless of time. The FPs were caused by a vertical sign board being detected as an intruder. Since these false positives were predicted more when the distance is farther away from camera, the precision decreases with an increase in the intruder distance. The i-LIDS-level evaluation does not reveal this information explicitly.

The edge-level results of Lohani2021+zscore at three distances also show that the recall increases rapidly as we move from 1 s towards 10 s. By visual inspecting, we found that these events consisted of intrusions where either the background of the video frame was similar to the intruder (camouflage), and/or the intruder was not standing straight, and/or there was a light flickering. Given more time (1 to 10 s), most of these intrusions at close and middle distances were detected, but the method was still not able to detect intrusions at a far distance due to the smaller object size. The three figures depict this phenomenon. Here, too, the precision scores remain almost constant with time. The FPs here were caused by different reasons, such as a flying bird, sudden light change and light flickering during night. These false positives were predicted more when the distance is farther away from camera; therefore, the precision decreases with an increase in the intruder distance. Again, the i-LIDS-level evaluation fails to reveal this information explicitly.

### 6.3. Overall Results for View 2

**Figure 14:**
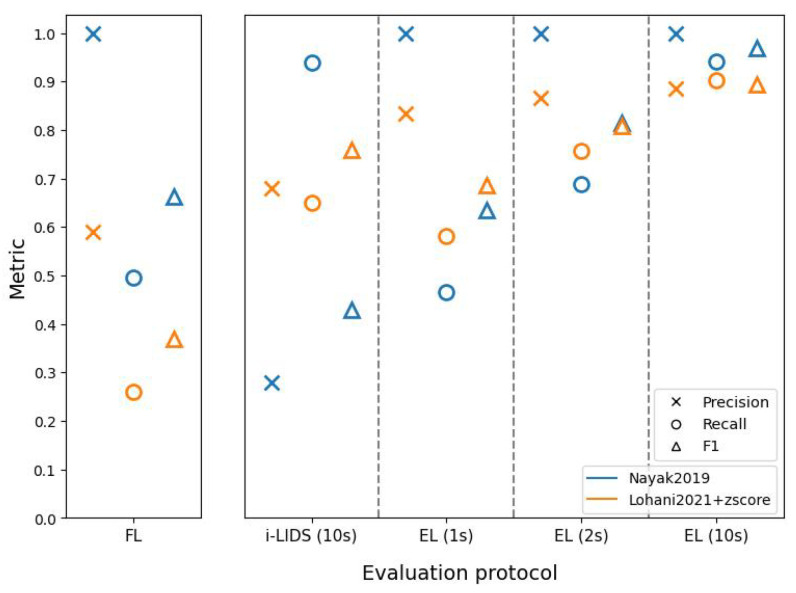
The results on view 2 of i-LIDS test set for two methods: Nayak et al. [47] (in blue) and Lohani et al. [6] with z-score (in orange). The abscissa represents three evaluation protocols: frame-level (FL) (on left subfigure), i-LIDS and edge-level (EL) (on right subfigure). i-LIDS is evaluated by default for 10 s whereas, for EL, we show results for 1, 2 and 10 s. The ordinate represents values of three metrics: precision, recall and $F_{1}$ score.

The results of view 2 are shown in Figure 14 (see Appendix B, Table A2 for tabular results). We can observe that Lohani2021+zscore has a similar trend of results like in view 1 in all evaluation protocols, but with a slight decrease in metric values. This slight decrease in values is due to an increase in FPs and FNs, which is linked to more videos of light fluctuation and intrusion at a far distance in monochrome videos of view 2. For Nayak2019, we obtain similar results to view 1 in frame-level and i-LIDS-level evaluation. In edge-level evaluation, we observe a perfect precision score of 1. This is because this method did not predict any false detection outside the intrusion interval in view 2. This perfect precision score augments the overall score of Nayak2019 in edge-level evaluation. This important detail of no false detection was not captured by the i-LIDS-level protocol and we observe a poor value of precision. We found similar observations to view 1 for the results of these two methods on view 2 with the intruder at different distances from the camera and, for sake of space, we provide these results in the Appendix A.

## 7. Discussion

It is clear that detecting intrusions should be evaluated at a higher level than frame-level. That is why almost all methods in the state of the art use i-LIDS evaluation [24,25,41]. However, the major problem with this evaluation is that it penalizes all alarms after 10 s, even if they are well within the intrusion interval. It is indeed too strict in counting FPs, and this hits the overall score negatively, regardless of the fact that the system has detected intrusion well within 10 s. To address the issue, we have proposed the edge-level evaluation. Here, we can parameterize the time after intrusion beginning for the evaluation. This helps in testing the system for different practical time settings and testing its robustness. More importantly, the alarms after a specified time are not counted as an FP if they are within the intrusion space. Thanks to this, we can evaluate a PIDS to check whether it has detected intrusions in the first few seconds without penalizing it for extra alarms.

These evaluation protocols further helped in understanding the two PIDSs. The method of Nayak et al. [47] had better frame-level scores than the method based on Lohani et al. [6], but this does not necessarily indicate that the former is a better PIDS. As we saw in edge-level scores, the latter method was better at detecting intrusion events and sending alarms quickly. In fact, EL(1 s) and EL(2 s) show that the latter method detected intrusions within 1 and 2 s, with a better performance than the former method. When we compare edge-level evaluation at 10 s with i-LIDS evaluation, we see that the former method has a very poor performance, while it performs similar to latter method at edge-level. This shows that i-LIDS evaluation is not able to capture this gain in performance. This ambiguity in scores shows that it is important to choose the right metric when evaluating a PIDS. The task here focuses on intrusion event and alarms; therefore, we must evaluate systems with a metric suitable for it. The edge-level evaluation should be used for this purpose with 1 or 2 s as time constraints in order to evaluate a PIDS in real-life conditions.

Although we have extensive real-life elements in the i-LIDS dataset, it has some major drawbacks. Firstly, it contains only humans as intruders, and not other objects, such as cars, bikes, trucks, etc. This is crucial because intrusions can also be caused by some other objects that the user has not thought of, as explained in Section 4.1.2, because $C_{na}$ is non-finite. Using a YOLO detector with supervised training on the VOC2012 dataset [79], the PIDS of Nayak et al. [47] explicitly defines $C_{na}$ as the single class human. Consequently, it gives very few false positives on the i-LIDS dataset, but there is a risk of omissions when this PIDS will protect sites with unexpected intruders, because it has a bias on the target class. On the contrary, the unsupervised PIDS of Lohani et al. [6] implicitly defines $C_{a}$ as all objects contained in the training set without intrusions. This approach seems to be more coherent with the definition of the PID task. The second crucial limitation is that we have only two views with very similar settings. This makes it easy for the algorithms (particularly supervised-learning-based) to learn the scene. A multi-view dataset would have added an additional difficulty in the PID task, allowing us to assess cross-view transfer learning. Therefore, we have a strong requirement of a new challenging dataset in this community, with multiple classes for intruders and several views.

In video surveillance, PID can be performed at different levels depending on the client requirements: on the edge device, on a server situated at the surveyed site or on the cloud. A PID running on the edge requires a computing device powered by a CPU or a GPU [47,59]. Most clients prefer the classical server-based analysis [4,6,22,24,25,41,48]. While these two solutions keep videos locally, the cloud analysis is not a widespread solution for the PID task because it requires a stable and secure network connection to stream videos in real-time, and it is not easy to ensure data privacy [2]. In our experiments, the methods have been tested on a single-camera dataset at the server. They can be deployed on the edge given appropriate devices, and they can also be scaled with more cameras given necessary computing resources and multi-camera datasets.

## 8. Conclusions

In this paper, we explored the task of perimeter intrusion detection in video surveillance. We first explained the typical PIDS pipeline and provided a review of major PIDS methods. We found that there are very few PIDSs that perform a full intrusion detection. We then provided a clear mathematical definition of an intrusion in an outdoor perimeter. In relation to the definition of intrusion, we revisited various existing evaluation protocols. We found that no existing evaluation protocol is suitable for the task and, therefore, we proposed a novel edge-level evaluation protocol. This protocol takes into account the real-life PIDS constraints, such as detecting intrusion in the first few seconds of its occurrence.

We also reviewed the existing PIDS datasets. We found that only the i-LIDS dataset is currently available for this task. This dataset was found to be challenging as it has different weather conditions, animals and intruder-approaching scenarios. It does have some major drawbacks, such as only person as the intrusion and just two camera views. Indeed, this community requires new datasets to develop and test intrusion detection systems.

Finally, we used two recent PIDSs to assess proposed definitions and evaluation protocols. We found that the frame-level evaluation does not give many details about the intrusion detection. The frames were poorly classified by both methods, giving us no finer details on which intrusion events were correctly detected. The i-LIDS evaluation protocol focused on evaluating intrusions at an event-level and thus helped in the better understanding of detections. However, it had a major drawback of penalizing system alarms as false detections, which made it difficult to compare the PIDSs. Finally, the edge-level evaluation overcame this drawback. It focused on evaluating the start of the intrusion event, which is completely coherent with the proposed intrusion definition and real-life constraints. As a result, the edge-level evaluation helped in the better understanding and comparison of the two PIDSs.

In the future work, we would like to further strengthen the unsupervised learning work for the PIDS. We hope that this work will result in motivating the community to propose methods with open-source codes and open-access challenging intrusion datasets in order to move reproducible research forward.

## Figures and Tables

**Table 1:** i-LIDS sterile zone dataset description.

				Number of Videos per Intrusion Count (Average Length in Minutes)						
**View**	**Set**	**Videos**	**Intrusions**	**0**	**1**	**10**	**13**	**15**	**17**	**31**
View 1	train	123	113	10 (29)	113 (3)	0	0	0	0	0
	test	17	113	10 (29)	0	2 (36)	1 (36)	1 (49)	2 (46)	1 (92)
View 2	train	113	103	10 (28)	103 (3)	0	0	0	0	0
	test	16	103	10 (28)	0	1 (37)	1 (36)	1 (49)	2 (46)	1 (92)

**Table 2:** PIDS reviewed by chronological order, where columns represent steps of the pipeline, along with model training needs and availability of source code. ✗ denotes unavailability of the step, whereas ✓ denotes that the step is available but not detailed.

Publication	Data Acquisition	Pre-Processing	Detection	Tracking	Post-Processing	Alarm	Model Training	Code Available
Buch and Velastin [41]	Visual camera	frame patches, FFT	inter-frame differencing	Kalman filter	✗	rule-based	none	✗
Vijverberg et al. [48]	Visual camera	✓	background subtraction	tracklet tracking	✗	✗	none	✗
Buch and Velastin [25]	Visual camera	frame patches, FFT	Gaussian background modeling	Kalman filter	✗	rule-based	none	✗
Vijverberg et al. [4]	Visual camera	✓	background subtraction	tracklet tracking	✓	rule-based	supervised	✗
Kim et al. [22]	Multi-camera: visual and thermal	resize, calibration, perimeter	2D CNN	particle filter	outside perimeter	rule-based	supervised	✗
Cermeno et al. [24]	Visual camera	perimeter	RGA	particle filter	object size rule	rule-based	none	✗
Nayak et al. [47]	Visual camera	perimeter	YOLO v2	SORT	✗	rule-based	supervised	✓
Lohani et al. [6]	Thermal camera	resize, normalization	3D ConvAutoencoder		✗	✗	unsupervised	✓

**Figure A1:**
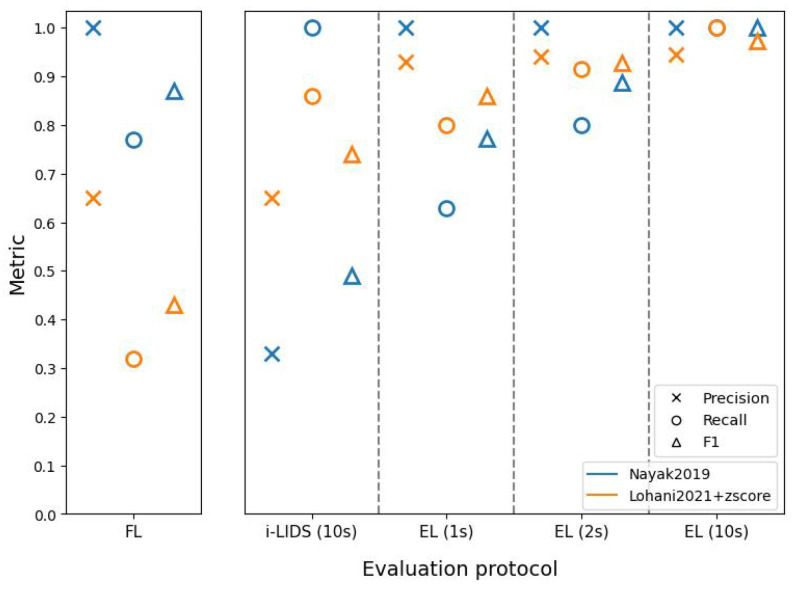
The results of two methods: [47] (in blue) and [6] with z-score (in orange), tested on view 2 of i-LIDS test set with intruder at close distance from camera. The abscissa represents three evaluation protocols: frame-level (FL) (on left subfigure), i-LIDS and edge-level (EL) (on right subfigure). i-LIDS is evaluated by default for 10 s whereas, for EL, we show results for 1, 2 and 10 s. The ordinate represents values of three metrics: precision, recall and $F_{1}$ score.

**Figure A2:**
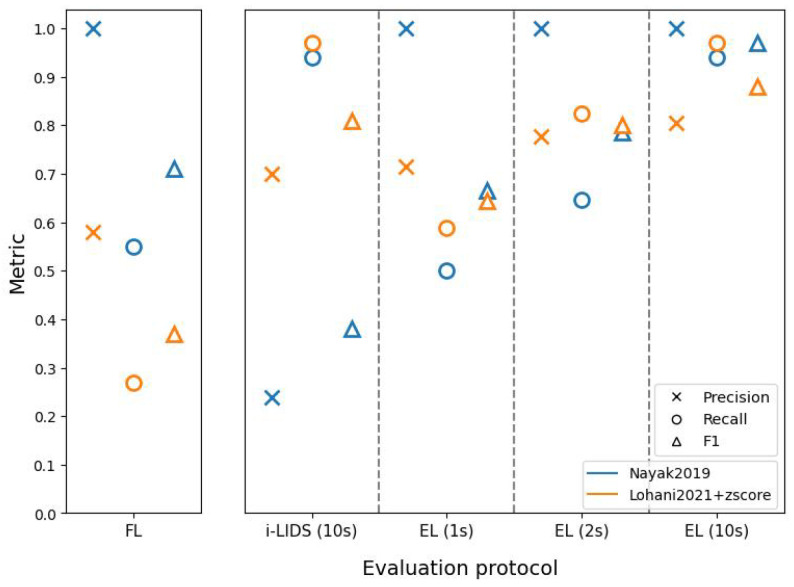
The results of two methods: [47] (in blue) and [6] with z-score (in orange), tested on view 2 of i-LIDS test set with intruder at mid distance from camera. The abscissa represents three evaluation protocols: frame-level (FL) (on left subfigure), i-LIDS and edge-level (EL) (on right subfigure). i-LIDS is evaluated by default for 10 s whereas, for EL, we show results for 1, 2 and 10 s. The ordinate represents values of three metrics: precision, recall and $F_{1}$ score.

**Figure A3:**
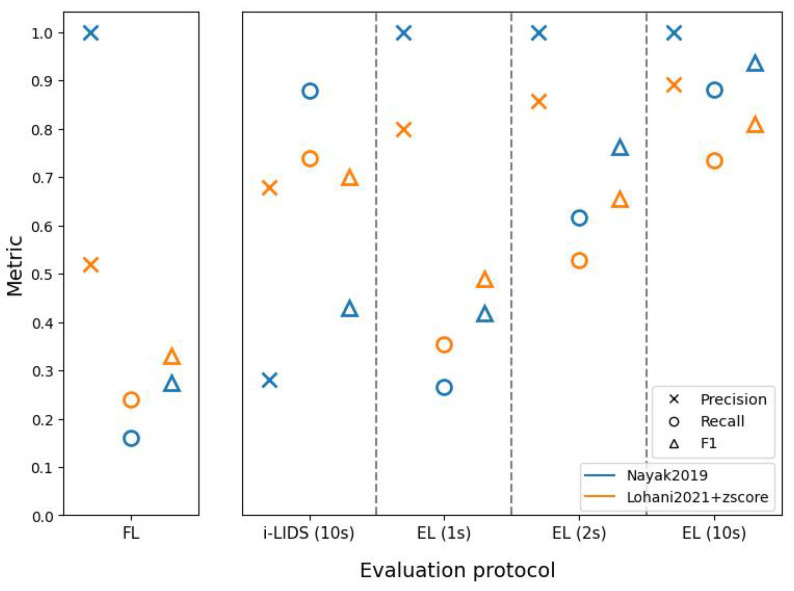
The results of two methods: [47] (in blue) and [6] with z-score (in orange), tested on view 2 of i-LIDS test set with intruder at far distance from camera. The abscissa represents three evaluation protocols: frame-level (FL) (on left subfigure), i-LIDS and edge-level (EL) (on right subfigure). i-LIDS is evaluated by default for 10 s whereas, for EL, we show results for 1, 2 and 10 s. The ordinate represents values of three metrics: precision, recall and $F_{1}$ score.

## Data Availability

Not applicable.

## Appendix A. Results for Intrusions at Different Distances from Camera for View 2

Figure A1, Figure A2 and Figure A3 present the results of the two methods at close, middle and far distances from the camera on the i-LIDS test dataset for view 2. These results are auto-explanatory as they follow a similar trend to the view 1 results (See Section 6.2.2).

## Appendix B. Results in Tabular Form

Table A1 and Table A2 show the results of the two methods on view 1 and view 2 of the i-LIDS test dataset through different evaluation protocols. These results are a tabular representation of Figure 9 and Figure 14, respectively. The columns FL, i-LIDS, EL (1 s) and so on represent different evaluation protocols, whereas P, R and $F_{1}$ denote precision, recall and $F_{1}$ scores.

**Table A1:** Results as percentage on view 1 of i-LIDS test set.

	FL			i-LIDS			EL (1 s)			EL (2 s)			EL (10 s)		
Method	P	R	$F_{1}$	P	R	$F_{1}$	P	R	$F_{1}$	P	R	$F_{1}$	P	R	$F_{1}$
Nayak2019	99	46	63	26	92	41	62	52	57	70	74	72	74	93	83
Lohani2021+zscore	64	27	38	80	82	81	90	73	80	92	88	90	92	91	92

**Table A2:** Results as percentage on view 2 of i-LIDS test set.

	FL			i-LIDS			EL (1 s)			EL (2 s)			EL (10 s)		
Method	P	R	$F_{1}$	P	R	$F_{1}$	P	R	$F_{1}$	P	R	$F_{1}$	P	R	$F_{1}$
Nayak2019	99	49	66	28	94	43	99	47	64	99	69	82	99	94	96
Lohani2021+zscore	59	26	37	68	85	76	83	58	69	87	76	81	89	90	89
